# Racial and Socioeconomic Disparity in Breast Cancer Mortality: A Systematic Review and Meta-Analysis

**DOI:** 10.3390/cancers17101641

**Published:** 2025-05-13

**Authors:** Helena Fiats Ribeiro, Fernando Castilho Pelloso, Beatriz Sousa da Fonseca, Camila Wohlenberg Camparoto, Maria Dalva de Barros Carvalho, Vlaudimir Dias Marques, Mariá Romanio Bitencourt, Kely Paviani Stevanato, Pedro Beraldo Borba, Deise Helena Pelloso Borghesan, Paulo Acácio Egger, Ana Carolina Jacinto Alarcão, Roberto Kenji Nakamura Cuman, Isabella Morais Tavares Huber, Márcia Edilaine Lopes Consolaro, Constanza Pujals, Carlos Laranjeira, Raíssa Bocchi Pedroso, Sandra Marisa Pelloso

**Affiliations:** 1 Health Sciences Center, State University of Maringá-UEM, Maringá 87020-900, Brazil; camila.wsouza1@gmail.com (C.W.C.); mdbcarvalho@gmail.com (M.D.d.B.C.); vdmarques@uem.br (V.D.M.); romanio.maria@gmail.com (M.R.B.); kelystevanato@gmail.com (K.P.S.); pa_egger@hotmail.com (P.A.E.); ana.alarcao@educaadventista.org.br (A.C.J.A.); rkncuman1@gmail.com (R.K.N.C.); constanza.pujals@gmail.com (C.P.); raissap@gmail.com (R.B.P.); smpelloso@uem.br (S.M.P.); 2 Municipal Health Department, Curitiba 80060-130, Brazil; fercaspell@ufpr.br; 3 Nurse Center, State University of Maringá—UEM, Maringá 87020-900, Brazil; beatriz.sousa.fonseca@hotmail.com; 4 Department of Medicine, UNIMAR—University of Marilia, São Paulo 17525-902, Brazil; bborbapedro@gmail.com; 5 Catholic College of Mato Grosso, Várzea Grande 78070-200, Brazil; prof.deisepelloso@uninga.edu.br; 6 Department of Medicine, Inga University Center—Uningá, Maringá 87035-510, Brazil; isabellatavares@hotmail.com; 7 Department of Biomedicine, State University of Maringá-UEM, Maringá 87020-900, Brazil; melconsolaro@uem.br; 8 School of Health Sciences, Polytechnic University of Leiria, Campus 2, Morro do Lena, Alto do Vieiro, Apartado 4137, 2411-901 Leiria, Portugal; carlos.laranjeira@ipleiria.pt; 9 Centre for Innovative Care and Health Technology (ciTechCare), Polytechnic University of Leiria, Campus 5, Rua das Olhalvas, 2414-016 Leiria, Portugal; 10 Comprehensive Health Research Centre (CHRC), University of Évora, 7000-801 Évora, Portugal

**Keywords:** socioeconomic disparity, culture-specific cancer outcomes, breast cancer

## Abstract

**Background/Objectives**: Breast cancer is one of the leading causes of female mortality worldwide, but significant racial and socioeconomic disparities persist in disease outcomes. This review aimed to analyze racial and socioeconomic inequalities in mortality and survival from breast cancer, identifying the impact of social risk factors on access to diagnosis and treatment. **Methods**: A systematic literature review and meta-analysis was performed following PRISMA guidelines. Eighteen studies published between 2014 and 2024 were included, with 11 contributing to the meta-analysis. Random-effect models were used to assess correlations between socioeconomic status, race, and clinical outcomes, including heterogeneity and publication bias analyses. **Results**: The strongest associations were observed between income, race, and breast cancer survival, with survival significantly worse among Black women and low-income populations (*p* < 0.001). Income also showed a strong positive correlation with clinical outcomes. In contrast, the overall effect on mortality was not statistically significant (*p* = 0.290), likely due to high heterogeneity across studies (I^2^ = 100%). These findings suggest that structural disparities in access to healthcare and early detection substantially affect survival rates. **Conclusions**: Racial and socioeconomic disparities in breast cancer outcomes remain critical public health challenges. Targeted policies to expand early diagnosis and ensure equitable access to effective treatment are essential to reduce these disparities and improve survival in underserved populations.

## Simple Summary

Breast cancer is a leading cause of death among women worldwide, but survival rates vary significantly depending on race and socioeconomic status. Women from lower-income backgrounds and minority populations often face barriers to early diagnosis and timely treatment, leading to worse clinical outcomes. This study examines racial and socioeconomic disparities in breast cancer mortality and survival, aiming to quantify the impact of these factors through a systematic review and meta-analysis. By analyzing data from multiple studies, we assess how access to healthcare, screening programs, and treatment availability influence survival rates. Our findings highlight the urgent need for policies that improve access to early detection and equitable treatment, particularly for underserved communities. Understanding these disparities can help guide healthcare interventions and research efforts to reduce inequalities in breast cancer outcomes and improve survival rates for vulnerable populations.

## 1. Introduction

With over 2.3 million new cases registered in 2022 [1] breast cancer is the most diagnosed cancer worldwide, 99% of which occur in women [2,3]. The World Health Organization (WHO) stated that breast cancer caused 670,000 deaths globally in 2022 [4]. Over 3 million cases and 1 million deaths from breast cancer are projected to occur yearly by 2040, which represents a global public health concern [2].

Several biological, environmental and social variables contribute to the development and progression of breast cancer [5,6]. Genetic factors (specifically family history), unhealthy lifestyle choices (e.g., diet, physical inactivity, smoking, drinking), environmental hazards (e.g., ionizing radiation), and social and psychological issues contribute to its prevalence [7,8]. Research has shown that hereditary factors and mutations account for 5–10% of breast cancer cases, while modifiable risk factors account for 20–30% [9].

Breast cancer mortality is intrinsically linked to racial and social inequities. Unfortunately, there is a significant disparity in mortality rates between different ethnic and socioeconomic groups, which reflects a clear inequality in access to health care and treatment outcomes. These racial inequities play a crucial role in breast cancer mortality. Studies have shown that women belonging to minority ethnic groups, such as Afro-descendants, Hispanics, and indigenous women have higher mortality rates compared to white women [10,11]. For example, in the United States, the five-year survival rate for non-Hispanic white women is approximately 91%, compared to 82% for Black women and 83% for Native American women [12]. Furthermore, Black women are 40% more likely to die from breast cancer than white women, despite having lower incidence rates [13]. These disparities reflect long-standing structural barriers in access to screening and timely treatment.

These inequalities can be attributed to a combination of factors, including differences in access to health services, lower quality of care, lower adherence to treatment, and diagnosis at more advanced stages of the disease. Women from low socioeconomic backgrounds, with lower educational levels and incomes, are more likely to face challenges in accessing health services and receive late diagnosis. These inequalities can be exacerbated by the lack of adequate health insurance, lack of transportation, language barriers, and lower awareness of the importance of early detection and regular follow-up [14].

Reducing disparities in breast cancer outcomes, early detection, and timely treatment depends on making relevant services available to everyone. The programs for reaching the underserved, however, must eliminate existing structural barriers, including but not limited to distance, costs, and poor health information. Available data support that structural factors, i.e., access to education, employment, and the health system, are not equal and have a significant effect on breast cancer outcomes, particularly in low- and middle-income countries [15,16]. Results that have been contrasted in studies across regions depict the ability of women in high-income countries to benefit from better coverage of screenings and access to treatment on time, women in resource-constrained settings often present with the disease at a later stage and few treatment options [16,17].

For instance, the WHO suggests that women in well-resourced areas who are 50 to 69 years old and at average risk for breast cancer undergo organized, population-based mammography screening every two years [18]. When resources are scarce—as they often are in low-resource areas where mammography screening is impractical or too expensive –, attention should instead be directed towards early detection by ensuring that women experiencing symptoms can get a proper diagnosis and treatment for their breast cancer as soon as possible [2].

Researchers have shown the need for breast cancer studies that address variables such as environmental and lifestyle factors; barriers preventing women from being diagnosed and treated, such as cultural taboos, geographic location, socioeconomic issues, and more effective methods of cancer prevention at the populational level, evaluating screening methods and techniques that meet women’s needs. In a study carried out by Yedjou et al. (2019), the authors report that racial and economic disparities persist, and one can identify and reduce these disparities. Although several other studies have shown the importance of knowing and verifying the impact of disparities on breast cancer mortality, to our knowledge, no studies have systematically reviewed the scientific literature to identify how these disparities affect the morbidity and mortality of women with breast cancer and, therefore, how to develop interventions to improve quality of life [19].

A solid grasp of the global patterns and variability in disease burden is essential for the success of these endeavors. Yedjou et al. (2019) highlighted that new strategies and approaches are needed to promote prevention, improve survival rates, reduce breast cancer mortality, and improve health outcomes. Understanding whether there is a relationship between breast cancer mortality and socioeconomic and geographical location variables is important to help build preventive strategies to reduce breast cancer mortality rates. Therefore, the primary aims of this systematic literature review and meta-analysis were to (a) summarize the findings of studies examining racial and socioeconomic inequalities in mortality and survival from breast cancer, and (b) determine the magnitude of the overall associations between breast cancer mortality and social risk factors on access to diagnosis and treatment through a meta-analysis. This information may offer direction to women aiming to mitigate their heightened risk of breast cancer.

## 2. Materials and Methods

### 2.1. Protocol and Registration

This study is a systematic review conducted according to the methodology of the Joanna Briggs Institute (JBI) [20], recognized for its rigorous and practical approach to reviews in the health area. The review was also conducted using the PRISMA (Preferred Reporting Items for Systematic Reviews and Meta-Analyses) and PRISMA-NMA guidelines [21]. This review is registered in PROSPERO (International Prospective Register of Systematic Reviews) under number CRD42024599149.

### 2.2. PICO(S) Criteria

The research design was structured based on the PICO(S) model, as follows: Population (P)—Women with breast cancer; Exposure/Indicator (I)—Analysis of mortality associated with breast cancer; Comparison (C)—Racial and social disparities in mortality; Outcome (O)—Identification of risk factors and impacts of screening strategies; and Type of Study (S)—Cross-sectional and longitudinal observational studies [22].

### 2.3. Search Strategy

In June 2024, a systematic literature search was conducted with the assistance of a research librarian. The search was conducted in the scientific databases PubMed, Embase, Scopus, Web of Science, Science Direct, SciELO, Biblioteca Virtual em Saúde (BVS), Cochrane Library and LILACS. Grey literature was also explored to broaden the scope of the results. The Medical Subject Heading (MeSH) terms “breast cancer”, “mortality”, “social health disparity” and “screening” were combined with Boolean operators and the search strategy was adapted to the specificities of each database. The search was conducted independently by two researchers and validated by the main author (Table 1).

### 2.4. Eligibility Criteria

The target was cross-sectional and longitudinal population-based observational studies with random sampling, published between 2014 to 2024. The year 2014 marks the publication of the World Health Organization’s position paper on mammography screening [23], which provided global guidance for breast cancer early detection strategies. Limiting the inclusion of studies published after this milestone ensures that the findings reflect contemporary practices, technologies, and health policy environments. Only studies with adult participants (≥18 years) and published in English, Portuguese or Spanish were considered. We included cross-sectional and longitudinal population-based studies because these observational designs are the most appropriate for investigating associations between social determinants and cancer outcomes at a population level. Randomized trials on racial and socioeconomic disparities are extremely limited, and other study designs (e.g., qualitative or case studies) do not allow for generalizable, quantitative comparisons of mortality or survival. Cross-sectional studies offered insight into prevalence and disparities, while longitudinal studies allowed evaluation of trends over time and survival estimates.

Studies conducted in specific populations, such as pregnant women and indigenous people, as well as systematic reviews, meta-analyses, dissertations, theses, technical reports, editorials and qualitative studies, were excluded. This decision was made because Indigenous populations often present unique sociocultural, geographic, and health system-related characteristics that distinguish them significantly from the general population. Including such studies in this meta-analysis could introduce substantial heterogeneity, as these groups face specific barriers to healthcare access and have different health outcomes that require a tailored analytical framework. Therefore, we opted to exclude them in order to preserve methodological consistency and comparability across studies.

### 2.5. Study Selection

The study selection occurred in three stages. Initially, six researchers (Group 1) screened the articles based on title and abstract. In case of disagreements, there was a discussion among the members or a final decision was made by the project coordinator. Subsequently, the selected articles were randomly distributed among the researchers for full reading and data extraction. The final stage involved an independent review by three experts (Group 2), who certified the final selection of studies.

### 2.6. Data Extraction and Analysis

The search results from each database were imported into Rayyan^®^ software (https://www.rayyan.ai, accessed on 14 August 2024) [24], a web-based tool designed to assist in the systematic review process, to streamline the review process. The extracted data included title, first author, year of publication, language, study objective, methodological design, collection site, sample size, mean age, socioeconomic variables and frequency of mammographic screening by age group and race/color.

The meta-analysis was conducted using a random-effects model, weighting the effects of each study by the inverse of its variance [25]. Heterogeneity was assessed using the I^2^ index and Cochran’s Q test, with values greater than 50% being considered indicative of moderate to high heterogeneity. Sensitivity analyses were performed using the robust Huber-White estimator and robust weighted variance meta-analysis. Statistical analyses were conducted using RStudio software, version 4.3.0.

Although the included studies differed in design (cross-sectional vs. longitudinal), all provided quantitative estimates of the association between sociodemographic variables and breast cancer mortality or survival. Given the small number of eligible studies and the use of a random-effects model, we chose to analyze the data jointly. Heterogeneity and publication bias were thoroughly assessed to ensure the robustness of the findings.

### 2.7. Assessment of Methodological Quality and Risk of Bias

Two researchers using the JBI checklist for prevalence studies and Crombie’s criteria for cross-sectional studies independently assessed the methodological quality of the studies. Nine items were considered, including sample adequacy, a detailed description of the setting and methodology, and measurement reliability. The risk of bias was analyzed using a funnel plot and Egger’s test. The assessment table is provided as Supplementary Materials (Table S1).

## 3. Results

### 3.1. General Presentation

**Figure 1:**
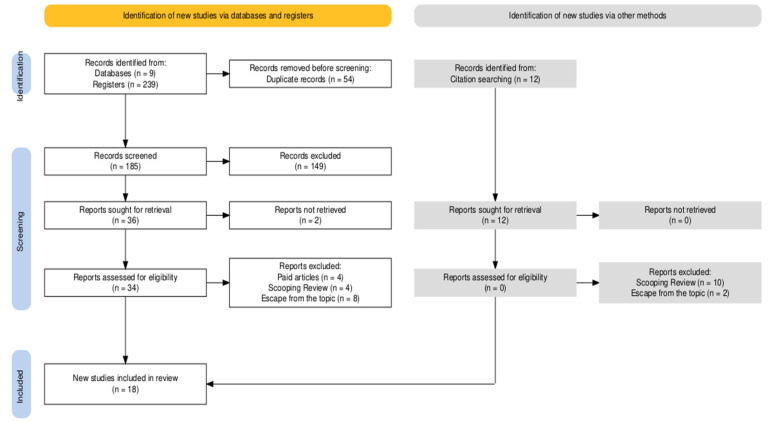
Flow diagram of the systematic review steps.

The initial search identified 239 articles, of which 54 were duplicates. After screening titles and abstracts, 38 articles were considered relevant for a full-text review. Of these, 16 articles were excluded because they did not meet the inclusion criteria. Therefore, 18 articles were included in the final sample and 11 participated in the meta-analysis (Figure 1).

After analyzing the 18 studies selected for this systematic review, the data were extracted and synthesized according to the research objectives. Regarding the geographical distribution of the studies, the majority (16) were carried out in the United States of America (USA), using large national databases such as SEER and the National Cancer Database [26,27,28,29,30,31,32,33,34,35,36,37,38,39,40,41]. Two studies were conducted in Brazil, investigating regional and socioeconomic inequalities in breast cancer outcomes [42,43]. No studies conducted exclusively in Europe or Asia were identified, although some multicenter articles included diverse populations [39,40].

Data for the studies were collected from the early 1990s to the late 2020s. Most reported annual data, while others reported aggregate trends over a decade [29,35]. The studies were cross-sectional analyses for retrospective cohorts and population-based registries based on national health systems [37,38]. One study assessed racial disparities exclusively, and the others assessed an interaction of differences in socioeconomic status, health insurance coverage, and regional disparities in breast cancer outcomes [31].

Methodological approaches varied, with eight studies using large national databases and others employing smaller-scale data from academic hospitals and regional health systems. The studies uniformly observed large heterogeneity in breast cancer mortality rates, diagnostic predictions, and treatment outcomes, highlighting the very complex interplay of biological, socioeconomic, and systemic factors that drive disparities.

Overall, the results of these studies emphasized that there is an urgent need for targeted public health and policy interventions to address the identified inequalities, especially among racial minorities and low-income populations. Table 2 demonstrates the individual characteristics of the studies included in the review.

### 3.2. Meta-Analysis

**Figure 2:**
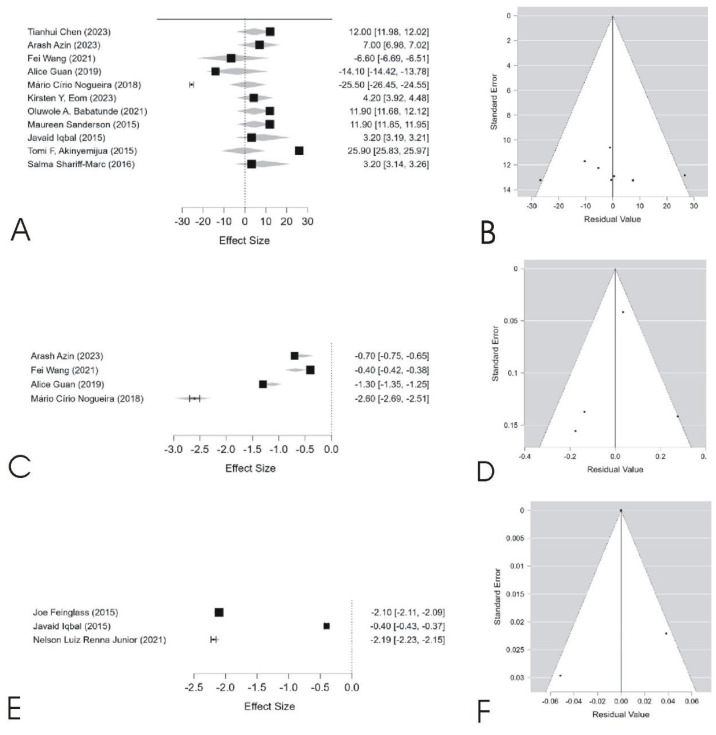
The figure presents the meta-analysis results for mortality (**A**,**B**), survival (**C**,**D**), and income (**E**,**F**). Forest plots (**A**,**C**,**E**) display the effect sizes and confidence intervals for each study, illustrating variability in estimates across different populations. Funnel plots (**B**,**D**,**F**) assess publication bias, where asymmetry suggests potential bias in mortality (**B**) and income (**F**), as confirmed by Egger’s test. High heterogeneity is observed in all outcomes, indicating significant variations among studies, which may be influenced by regional, socioeconomic, and methodological differences. Original figure created by the authors based on data included in the present study.

The data presented in Table 3 and Figure 2 indicate that mortality did not exhibit a significant combined effect (*p* = 0.290, coefficient = −0.192), despite high heterogeneity (I^2^ = 100%, *p* < 0.001), suggesting substantial variations across studies. These differences may be influenced by factors such as regional disparities, healthcare policies, and population characteristics. Additionally, Egger’s test (*p* = 0.003) suggests potential publication bias, supported by the asymmetry in the funnel plot (Figure 2B).

In contrast, the survival analysis revealed a significant positive effect (*p* < 0.001, coefficient = 5.596, 95% CI: 4.377 to 6.815), with high heterogeneity (I^2^ = 98.6%, *p* < 0.001). Although the corresponding funnel plot (Figure 2D) appears relatively symmetrical, Egger’s test (*p* = 0.006) indicates mild publication bias. Similarly, income showed a significant association with outcomes (*p* < 0.001, coefficient = 5.010, 95% CI: 4.757 to 5.263), with notable heterogeneity (I^2^ = 84.7%). The funnel plot (Figure 2F) suggests substantial asymmetry, confirmed by Egger’s test (*p* < 0.001). These findings highlight the influence of socioeconomic and demographic factors on the analyzed outcomes and reinforce the need for careful interpretation, particularly in the presence of high heterogeneity and potential bias.

The high heterogeneity observed in the meta-analysis (I^2^ = 100% for mortality, I^2^ = 98.6% for survival) suggests that racial and socioeconomic disparities in breast cancer outcomes vary across different contexts. These variations may be attributed to differences in healthcare systems, screening policies, and population characteristics. Although some studies report survival improvements among Black women, heterogeneity remains high, indicating persistent structural challenges that hinder full equity.

While subgroup analyses could help identify sources of heterogeneity, the limited number of eligible studies (n = 11) reduced the feasibility of stratified meta-analyses. We acknowledge this as a limitation and suggest that future reviews explore larger datasets or focus on specific study designs to enable meta-regression or subgroup comparisons.

In addition to the aggregated effects presented above, Table 4 provides a study-by-study summary of the 11 articles included in the meta-analysis, specifying the outcome assessed, the main sociodemographic variables evaluated, and whether the associations were statistically significant.

For each study included in the meta-analysis, key information was systematically extracted, including sample size, outcomes assessed, sociodemographic variables analyzed, effect size (ES), standard error (SE), and statistical significance. Effect sizes and corresponding standard errors were directly collected from the results reported by the original authors, considering adjusted estimates (e.g., Odds Ratio, Hazard Ratio, or regression coefficients) whenever available. When the standard error was not explicitly provided, it was calculated based on the confidence intervals reported, following standard statistical procedures. Statistical significance was recorded according to the criteria adopted by each study, with *p*-values < 0.05 considered significant.

### 3.3. Methodological Quality Assessment

The studies included in this review were classified according to their methodological design, using the Joanna Briggs Institute’s (JBI) criteria applied to prevalence studies and Crombie’s criteria used for cross-sectional studies. The methodological assessment indicated that, in most prevalence studies, quality criteria were adequately met, particularly regarding sample representativeness, appropriate recruitment, and the use of validated measures. However, some limitations were identified, especially concerning the detailed description of the study setting and methodology, as well as the lack of control for confounding factors, which may compromise the reliability of the findings.

Overall, the methodological quality of the included studies was classified as moderate to high, with some specific limitations. Nevertheless, the presence of studies with low sample representativeness and insufficient control of confounding variables should be considered when interpreting the findings of this systematic review

### 3.4. Bias Risk Assessment

**Figure 3:**
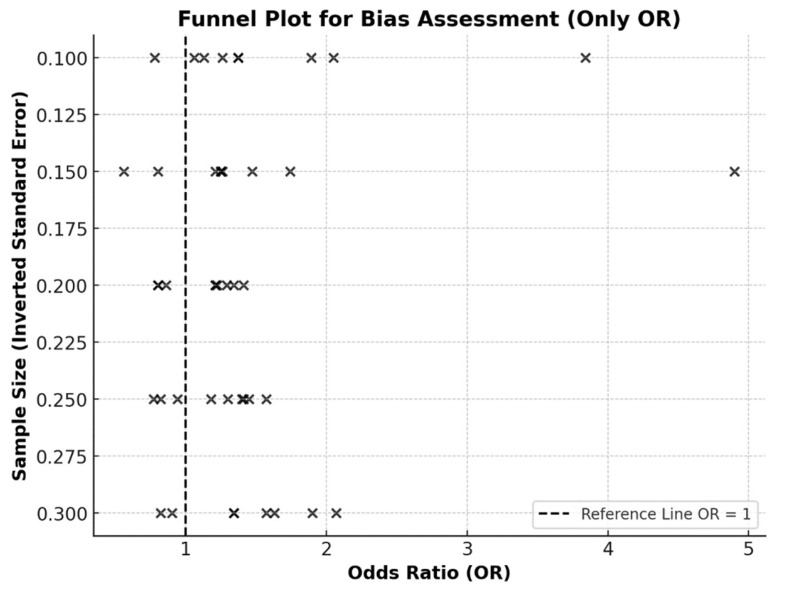
Funnel plot (inverted, or “Christmas tree” shape) assessing potential bias in studies reporting odds ratios (OR). Each “×” denotes a single study. The dashed vertical line represents the null effect (OR = 1).

To assess the risk of bias, an inverted funnel plot and Egger’s test were used [44,45]. The funnel plot (Figure 3) visually suggests a relatively balanced distribution of studies, indicating no strong publication bias. Egger’s test (*p* = 0.832) further confirmed the absence of publication bias (*p* > 0.05), suggesting that the findings of this review are robust and reliable.

### 3.5. Summarized Results

Differences in access to healthcare services and mammographic screening significantly impact clinical outcomes among socioeconomic groups. Patients with private health insurance are more likely to undergo regular mammograms and receive an early diagnosis compared to those with public insurance [39]. However, paradoxically, privately insured patients also exhibit a higher risk of diagnostic delays compared to those in the public healthcare system [35], potentially due to differences in referral protocols, waiting times, and treatment coverage.

Regular mammographic screening is associated with a significant reduction in breast cancer mortality, with studies indicating up to a 45% mortality reduction [40]. Moreover, shorter screening intervals have been linked to reduced overall mortality (adjusted OR = 0.57; 95% CI: 0.36–0.89; *p* = 0.013) [27]. However, low-income women continue to have reduced access to regular screenings, contributing to delayed diagnoses and increased mortality rates.

Socioeconomic factors such as income, education, and housing conditions strongly influence breast cancer outcomes [43]. Women with lower educational attainment exhibit higher mortality rates even after adjusting for clinical variables (HR = 1.27; 95% CI: 1.24–1.31) [42]. Patients in socioeconomically disadvantaged communities also face barriers such as limited access to advanced medical technologies and reduced social support, directly impacting survival [41]. These findings highlight the need for targeted public policies to ensure equitable access to screening and treatment, minimizing socioeconomic disparities.

Finally, the methodological quality assessment revealed that while most studies employed appropriate statistical methods, approximately 40% did not adequately justify sample size, and 35% had methodological deficiencies that may have influenced results. However, the absence of publication bias, as confirmed by Egger’s test (*p* = 0.832), suggests that the findings of this review are reliable and robust.

## 4. Discussion

Although breast cancer mortality has been extensively studied worldwide, gaps remain regarding racial and socioeconomic disparities associated with the disease. Our meta-analysis reinforces that socioeconomic and racial factors remain decisive in determining breast cancer outcomes, particularly in relation to survival rates. The high degree of heterogeneity found among the studies analyzed indicates significant variations in demographic characteristics, methodological differences, and access to healthcare systems.

According to Ahmed [46], barriers to accessing mammography screening and early treatment are closely linked to socioeconomic and racial factors. Similarly, a meta-analysis by Silva et al. [15] found that women in low- and middle-income countries experience higher mortality rates due to late diagnosis and a lack of screening infrastructure. These findings suggest that access to healthcare services significantly influences disparities in breast cancer prognosis.

The discrepancies in survival rates among women with breast cancer reflect not only biological tumor behavior but also the impact of social determinants such as access to healthcare services and socioeconomic status. Newman et al. [13] emphasize the importance of public policies aimed at eliminating barriers faced by low-income African-American and Hispanic women, ensuring early screening, appropriate treatments, and strategies tailored to vulnerable populations. Additionally, educational programs, free screenings funded by non-commercial sources, and initiatives promoting equitable distribution of modern evidence-based treatments have been proposed to reduce these disparities.

Targeted intervention programs have demonstrated positive effects on survival among vulnerable groups. According to Grant et al. [47], while such programs improve outcomes, uneven coverage of screening services sustains gaps in late-stage diagnoses. Yedjou et al. [19] further suggest that awareness-raising and social support programs can help reduce inequalities in disadvantaged populations, highlighting the need for more comprehensive interventions.

There is a strong correlation between low income and more advanced clinical stages of breast cancer. Women with lower socioeconomic status have limited access to preventive screenings, early diagnosis, and guideline-recommended drug treatments, which contributes to disease progression and increased mortality. Our meta-analysis confirms these associations, adding pooled statistical support to the literature. Opia [48] observed that economic interventions aimed at expanding access to screening and treatment are associated with reduced inequality. This is supported by Pearson [49], who emphasizes that financial and geographic barriers continue to limit timely access to screening, particularly for women relying on underfunded public health systems.

### Study Limitations

The included studies were assessed using different methodological criteria: the JBI Checklist for prevalence studies and Crombie’s Criteria for cross-sectional studies. While this approach ensured a systematic and structured analysis, differences in methodological designs may have influenced the findings. Notably, variations in sample size justification and inadequate control of confounding factors in some studies contributed to the observed heterogeneity. One limitation is the combination of different study designs (cross-sectional and longitudinal), which may introduce heterogeneity. However, this was mitigated by the use of a random-effects model and sensitivity analyses. Additionally, the lack of standardization in analyzed variables impacted the consolidated interpretation of findings. Differences in statistical methods, inclusion and exclusion criteria, and outcome measurement approaches pose challenges for generalizing conclusions. Approximately 45% of the studies did not provide clear information on response rates or sample attrition management, potentially introducing bias.

Despite these limitations, the rigorous selection and methodological assessment process—conducted independently and repeatedly—enhances the reliability of this meta-analysis. This comprehensive and methodologically robust approach provides valuable insights into the impact of socioeconomic and racial disparities on breast cancer mortality.

Future research should address these limitations through methodological standardization, more detailed sample size justification, stricter control of confounding factors, and inclusion of grey literature. These efforts will ensure more precise and comparable analyses, further elucidating the impact of inequalities on breast cancer outcomes. Further studies should also consider using mixed-methods approaches, including qualitative analyses, to explore how social, cultural, and psychological dimensions shape access to cancer care and influence outcomes. Such approaches can offer a deeper understanding of the lived experiences behind the statistical disparities. Additionally, there is a need to include evidence from underrepresented regions, particularly in Asia and Africa, where socioeconomic and racial dynamics may differ substantially. Expanding geographic representation will enhance the generalizability of findings and support the development of more globally inclusive health policies.

## 5. Conclusions

This systematic review and meta-analysis highlighted that racial and socioeconomic disparities continue to play a major role in breast cancer outcomes, especially for African American, Hispanic populations, and those belonging to low-income groups. While such inequalities have been reported in previous studies, few have synthesised comparable quantitative data on death and survival, which indicate high heterogeneity at the population and regional levels. The findings bring out the need for public policies to ensure access and equal rights, to have screening done as early as possible, and to diagnose and treat breast cancer. Actions should include having government-funded programs for screening on a population basis, educating vulnerable communities, and ensuring that there is a fair allocation of evidence-based interventions. On top of this, the current disparities in cancer care show the need for an international collaborative network to cut gaps in equitable access and early detection. Health policies must reinforce health systems with financial support for cancer control programs and ensure that every patient has access to high-quality cancer services. Only through shared world plans can we truly achieve fair results in breast cancer prevention and care.

## Figures and Tables

**Table 1:** Search Strategies Conducted in the Databases.

Database	Search Strategy
PUBMED	((((breast cancer) AND (mortality)) AND (screening)) AND (social disparity)) AND (racial disparity)
EMBASE	(mortality), (screening), (breast cancer), (social determinants of health), (disparity)
Virtual Health Library (BVS)	(breast cancer) AND (mortality) AND (screening) AND (social disparity) AND (racial disparity)
LILACS	(breast cancer) AND (mortality) AND (screening) AND (social disparity) AND (racial disparity)
WEB OF SCIENCE	(breast cancer) AND (mortality) AND (screening) AND (social disparity) AND (racial disparity)
MEDLINE	(breast cancer) AND (mortality) AND (screening) AND (social disparity) AND (racial disparity)
SCIENCEDIRECT	(breast cancer) AND (mortality) AND (screening) AND (social disparity) AND (racial disparity)
COCHRANE	(breast cancer) AND (mortality) AND (screening) AND (disparities)

**Table 2:** General characteristics of the articles included in the review.

Citation (First Author et al., Year)	Country	Sample Number	Brief Conclusion
Anderson et al., 2023	USA	2.176	Geographic disparities influence breast cancer mortality, with access to treatment a critical determinant.
Chen et al., 2023	USA	415.277	Advanced diagnostic methods reduce disparities, but rural populations still face higher mortality rates.
Azin et al., 2023	USA	382.975	Advanced tumor staging highlights the role of socioeconomic status in breast cancer survival rates.
Fei Wang et al., 2021	USA	78.708	Urban populations have better outcomes due to better access to diagnostic and treatment facilities.
Guan et al., 2019	USA	5.622	Disparities in genetic screening contribute to differential outcomes in minority groups.
Nogueira et al., 2018	Brazil	481	Regional inequalities exacerbate disparities in breast cancer diagnosis stage and mortality.
Li Tao et al., 2016	USA	103.498	Hormone receptor status (ER+/PR+) significantly affects survival, particularly in low socioeconomic status groups.
Boyko et al., 2024	USA	907	Late screening and late-stage diagnosis are associated with higher mortality, even in safety-net hospitals.
Eom et al., 2023	USA	2.284	Targeted interventions in underserved regions reduce late-stage diagnoses and mortality.
Babatunde et al., 2021	USA	3.286	Late diagnosis and limited access to treatment are key factors leading to poor outcomes in low-income settings.
Renna Junior et al., 2021	Brazil	2.045 in Aracaju, 7.872 in Curitiba	Inequalities in access to treatment are important factors in breast cancer mortality in deprived regions.
Feinglass et al., 2015	USA	582.396	Socioeconomic status has a strong impact on mortality, even after adjustment for stage and clinical characteristics.
Sanderson et al., 2015	USA	64.384	Regular mammography reduces mortality by up to 45%, but disparities persist among black women.
Iqbal et al., 2015	USA	373.563	Black women are less likely to be diagnosed at stage I and have higher mortality, even for small tumors.
Akinyemiju et al., 2016	USA	67.084	Black women have higher hospital mortality and post-surgical complications compared to white women.
John et al., 2021	USA	10.366	ER+/PR+ breast cancer mortality is significantly higher in African American women due to socioeconomic status and hospital-type.
Akinyemijua et al., 2015	USA	71.156	Black women face higher hospital mortality and receive fewer mastectomies compared to white women.
Shariff-Marc et al., 2016	USA	9.372	Racial/ethnic disparities in mortality after breast cancer diagnosis highlight the influence of socioeconomic status and race.

**Table 3:** Meta-Analysis Results for Mortality, Survival, and Income.

Outcome	Coefficient (Estimate)	Standard Error	z-Value	*p*-Value	CI 95% (Lower Upper)	I^2^ (%)	τ^2^	Egger’s Test (*p*)
Mortality	−0.192	0.181	−1.059	0.29	−0.548 to 0.163	100	195.272	0.003
Survival	5.596	0.622	9.004	<0.001	4.377 to 6.815	98.604	0.032	0.006
Income	5.010	0,129	38.755	<0.001	4.757 to 5.263	84.708	0.001	<0.001

**Table 4:** Summary of key data extracted from the 11 studies included in the Meta-Analysis.

Author (Year)	Country	Sample Size (n)	Outcome Assessed	Effect Size (ES)	SE	Sociodemographic Variables	Statistically Significant?
Tianhui Chen (2023)	USA	415,277	Mortality	12.0	0.0101	Race, income	Yes
Arash Azin (2023)	USA	382,975	Mortality	7.0	0.0083	SES	Yes
Fei Wang (2021)	USA	78,708	Survival	−6.6	0.0458	Urban vs. rural residence	Yes
Alice Guan (2019)	USA	5622	Survival	−14.1	0.1657	Genetic ancestry	Yes
Mário C. Nogueira (2018)	Brazil	481	Mortality	−25.5	0.4858	Region, income	No
Kirsten Y. Eom (2023)	USA	2284	Survival	4.2	0.1438	Race, insurance	Yes
Oluwole A. Babatunde (2021)	USA	3286	Mortality	11.9	0.1143	Insurance, income	Yes
Maureen Sanderson (2015)	USA	64,384	Mortality	11.9	0.0258	Race, mammography	Yes
Javaid Iqbal (2015)	USA	373,563	Mortality	3.2	0.005	Race, stage at diagnosis	Yes
Tomi F. Akinyemijua (2015)	USA	71,156	Mortality	25.9	0.0342	SES, surgical treatment	Yes
Salma Shariff-Marc (2016)	USA	9372	Mortality	3.2	0.0313	Race/ethnicity, SES	Yes

Abbreviations: ES = Effect Size; SE = Standard Error; SES = Socioeconomic Status.

## Data Availability

This study is based on a systematic review and meta-analysis of previously published research. All data used were extracted from peer-reviewed articles indexed in databases such as PubMed, Scopus, and Web of Science. The list of included studies is provided in Table 2 and in the References section.

## Supplementary Materials

The following supporting information can be downloaded at: https://www.mdpi.com/article/10.3390/cancers17101641/s1, Table S1: Methodological Quality of Prevalence Studies (JBI Checklist).

## References

[1] WHO (2025). Breast Cancer Cases and Deaths Are Projected to Rise Globally. https://www.iarc.who.int/wp-content/uploads/2025/02/pr361_E.pdf.

[2] Arnold M., Morgan E., Rumgay H., Mafra A., Singh D., Laversanne M., Vignat J., Gralow J.R., Cardoso F., Siesling S. (2022). Current and future burden of breast cancer: Global statistics for 2020 and 2040. Breast.

[3] Sung H., Ferlay J., Siegel R.L., Laversanne M., Soerjomataram I., Jemal A. (2021). Global cancer statistics 2020: GLOBOCAN estimates of incidence and mortality worldwide for 36 cancers in 185 countries. CA Cancer J. Clin..

[4] WHO (2024). Breast Cancer. https://www.who.int/news-room/fact-sheets/detail/breast-cancer.

[5] Aizaz M., Khan M., Khan F.I., Ahmad S., Obeagu E.I. (2023). Burden of breast cancer: Developing countries perspective. Int. J. Innov. Appl. Res..

[6] Obeagu E.I., Babar Q., Vincent C.C., Udenze C.L., Eze R., Okafor C.J., Ifionu B.I., Amaeze A.A., Amaeze F.N. (2021). Therapeutic targets in breast cancer signaling: A review. J. Pharm. Res. Int..

[7] Løyland B., Sandbekken I.H., Grov E.K., Utne I. (2024). Causes and Risk Factors of Breast Cancer, What Do We Know for Sure? An Evidence Synthesis of Systematic Reviews and Meta-Analyses. Cancers.

[8] Cohen S.Y., Stoll C.R., Anandarajah A., Doering M., Colditz G.A. (2023). Modifiable risk factors in women at high risk of breast cancer: A systematic review. Breast Cancer Res..

[9] Sun Y.S., Zhao Z., Yang Z.N., Xu F., Lu H.J., Zhu Z.Y., Shi W., Jiang J., Yao P.P., Zhu H.P. (2017). Risk factors and preventions of breast cancer. Int. J. Biol. Sci..

[10] Hines R.B., Johnson A.M., Lee E., Erickson S., Rahman S.M.M. (2021). Trends in breast cancer survival by race-ethnicity in Florida, 1990–2015. Cancer Epidemiol. Biomark. Prev..

[11] Miller B.C., Bowers J.M., Payne J.B., Moyer A. (2019). Barriers to mammography screening among racial and ethnic minority women. Soc. Sci. Med..

[12] DeSantis C.E., Ma J., Gaudet M.M., Newman L.A., Miller K.D., Sauer A.G., Jemal A., Siegel R.L. (2019). Breast cancer statistics, 2019. CA Cancer J. Clin..

[13] Newman L.A. (2015). Disparities in breast cancer and African ancestry: A global perspective. Breast J..

[14] Ribeiro H.F., Carvalho M.D.d.B., Pelloso F.C., Santos L.d., Silva M.d.A.P., Stevanato K.P., Borghesan D.H.P., Romani I., Marques V.D., de Freitas K.M.S. (2023). Maternal Risk Factors Associated with Negative COVID-19 Outcomes and Their Relation to Socioeconomic Indicators in Brazil. Healthcare.

[15] E Silva J.D.D., Pedroso R.B., Pelloso F.C., Carvalho M.D.B., Santos T.D.S., Dutra A.C., Stevanato K.P., Marques V.D., De Andrade L., Araújo D.C.M. (2024). Mortality of Young Women due to Breast Cancer in Low, Middle and High-Income Countries: Systematic Literature Review and Meta-Analysis. Asian Pac. J. Cancer Prev..

[16] Anampa-Guzmán A., Acevedo F., Partridge A.H., Alfano C.M., Nekhlyudov L. (2021). Cancer Survivorship in Latin America: Current Status and Opportunities. JCO Glob. Oncol..

[17] Unger-Saldaña K. (2014). Challenges to the early diagnosis and treatment of breast cancer in developing countries. World J. Clin. Oncol..

[18] WHO (2014). Position Paper on Mammography Screening. https://www.who.int/publications/i/item/9789241507936.

[19] Yedjou C.G., Sims J.N., Miele L., Abusamaan M.S., Payton M., Tchounwou P.B. (2019). Health and racial disparity in breast cancer. Adv. Exp. Med. Biol..

[20] Joanna Briggs Institute (2018). Annual Report 2018.

[21] Page M.J., McKenzie J.E., Bossuyt P.M., Boutron I., Hoffmann T.C., Mulrow C.D., Moher D., Shamseer L., Tetzlaff J.M., Akl E.A. (2021). The PRISMA 2020 statement: An updated guideline for reporting systematic reviews. BMJ.

[22] Schardt C., Adams M.B., Owens T., Keitz S., Fontelo P. (2007). Utilization of the PICO framework to improve searching PubMed for clinical questions. BMC Med. Inform. Decis. Mak..

[23] World Health Organization (2014). WHO Position Paper on Mammography Screening.

[24] Hammady M., Fedorowicz Z., Elmagarmid A. (2016). Rayyan—A web and mobile app for systematic reviews. Syst. Rev..

[25] Borenstein M., Hedges L.V., Higgins J.P.T., Rothstein H.R. (2009). Introduction to Meta-Analysis.

[26] Anderson T., Herrera D., Mireku F., Barner K., Kokkinakis A., Dao H., Webber A., Merida A.D., Gallo T., Pierobon M. (2023). Geographical variation in social determinants of female breast cancer mortality across US counties. JAMA Netw. Open.

[27] Akinyemiju T., Sakhuja S., Vin Raviv N. (2018). Racial and socio-economic disparities in breast cancer hospitalization outcomes by insurance status. Ethn. Health.

[28] Feinglass J., Rydzewski N., Yang A. (2015). The socioeconomic gradient in all-cause mortality for women with breast cancer: Findings from the 1998 to 2006 National Cancer Data Base with follow-up through 2011. Ann. Epidemiol..

[29] John E.M., McGuire V., Kurian A.W., Koo J., Shariff-Marco S., Gomez S.L., Cheng I., Keegan T.H.M., Kwan M.L., Bernstein L. (2021). Racial/ethnic disparities in survival after breast cancer diagnosis by estrogen and progesterone receptor status: A pooled analysis. Cancer Epidemiol. Biomark. Prev..

[30] Boyko A., Qureshi M.M., Fishman M.D.C., Slanetz P.J. (2024). Predictors of breast cancer outcome in a cohort of women seeking care at a safety net hospital. Acad. Radiol..

[31] Guan A., Lichtensztajn D., Oh D., Jain J., Tao L., Hiatt R.A., Gomez S.L., Fejerman L. (2020). Breast cancer in San Francisco: Disentangling disparities at the neighborhood level. Cancer Epidemiol. Biomark. Prev..

[32] Azin A., Tahmasebi H., Brar A., Azin S., Ko G., Covelli A., Cil T. (2023). Racial, ethnic and socioeconomic disparities in diagnosis, treatment, and survival of patients with breast cancer. Am. J. Surg..

[33] Tao L., Gomez S.L., Keegan T.H.M., Kurian A.W., Clarke C.A. (2015). Breast cancer mortality in African-American and non-Hispanic white women by molecular subtype and stage at diagnosis: A population-based study. Cancer Epidemiol. Biomark. Prev..

[34] Shariff-Marco S., Yang J., John E.M., Kurian A.W., Cheng I., Leung R., Koo J., Monroe K.R., Henderson B.E., Bernstein L. (2015). Intersection of race/ethnicity and socioeconomic status in mortality after breast cancer. J. Community Health.

[35] Babatunde O.A., Eberth J.M., Felder T., Moran R., Truman S., Hebert J.R., Zhang J., Adams S.A. (2021). Social determinants of racial disparities in breast cancer mortality among Black and White women. J. Racial Ethn. Health Disparities.

[36] Wang F., Zheng W., Bailey C.E., Mayer I.A., Pietenpol J.A., Shu X.O. (2021). Racial/ethnic disparities in all-cause mortality among patients diagnosed with triple-negative breast cancer. Cancer Res..

[37] Iqbal J., Ginsburg O., Rochon P.A., Sun P., Narod S.A. (2015). Differences in breast cancer stage at diagnosis and cancer-specific survival by race and ethnicity in the United States. JAMA.

[38] Sanderson M., Levine R.S., Fadden M.K., Kilbourne B., Pisu M., Cain V., Husaini B.A., Langston M., Gittner L., Zoorob R. (2015). Mammography screening among the elderly: A research challenge. Am. J. Med..

[39] Chen T., Kharazmi E., Fallah M. (2023). Race and ethnicity-adjusted age recommendation for initiating breast cancer screening. JAMA Netw. Open.

[40] Eom K.Y., Berg K.A., Joseph N.E., Runner K., Tarabichi Y., Khiyami A., Perzynski A.T., Sossey-Alaoui K. (2023). Neighborhood and racial influences on triple negative breast cancer: Evidence from Northeast Ohio. Breast Cancer Res. Treat..

[41] Akinyemiju T.F., Vin-Raviv N., Chavez-Yenter D., Zhao X., Budhwani H. (2015). Race/ethnicity and socio-economic differences in breast cancer surgery outcomes. Cancer Epidemiol..

[42] Nogueira M.C., Guerra M.R., Cintra J.R.D., Corrêa C.S.L., Fayer V.A., Bustamante-Teixeira M.T. (2018). Disparidade racial na sobrevida de 10 anos no câncer de mama: Uma análise de mediação usando abordagem de respostas potenciais. Cad. Saúde Pública..

[43] Junior N.L.R., Lima C.A., Laporte C.A., Coleman M.P., Silva G.A.E. (2021). Ethnic, racial and socioeconomic disparities in breast cancer survival in two Brazilian capitals between 1996 and 2012. Cancer Epidemiol..

[44] Egger M., Smith G.D., Schneider M., Minder C. (1997). Bias in meta-analysis detected by a simple, graphical test. BMJ.

[45] Zhou Y., Huang A., Hattori S. (2023). A likelihood-based sensitivity analysis for publication bias on the summary receiver operating characteristic in meta-analysis of diagnostic test accuracy. Stat. Med..

[46] Ahmed A.T., Welch B.T., Brinjikji W., Farah W.H., Henrichsen T.L., Murad M.H., Knudsen J.M. (2017). Racial disparities in screening mammography in the United States: A systematic review and meta-analysis. J. Am. Coll. Radiol..

[47] Grant S.J., Yanguela J., Odebunmi O., Grimshaw A.A., Giri S., Wheeler S.B. (2024). Systematic review of interventions addressing racial and ethnic disparities in cancer care and health outcomes. J. Clin. Oncol..

[48] Opia F.N., Matthew K.A. (2025). Socioeconomic disparities in breast cancer care: Addressing global challenges in oncology outcomes. Int. J. Comput. Appl. Technol. Res..

[49] Pearson S.A., Taylor S., Marsden A., Zdenkowski N., Bates N., McNeil C., Nair B., Wyld D., Stockler M.R., Kiely B.E. (2024). Geographic and sociodemographic access to systemic anticancer therapies for secondary breast cancer: A systematic review. Syst. Rev..

