# Supplementary material for: Racial and Socioeconomic Disparity in Breast Cancer Mortality: A Systematic Review and Meta-Analysis
Source: Cancers (Basel). 2025 May 13;17(10):1641. doi: 10.3390/cancers17101641 (PMC12109952; doi:10.3390/cancers17101641)
Supplement: Supplementary file 1 [file cancers-17-01641-s001.zip › cancers-3547361-supplementary.pdf]

Table S1. Methodological Quality of Prevalence Studies (JBI Checklist).

Author (Year)	Was the sampling frame appropriate to address the target population	Were study participants sampled appropriately	Was the sample size adequate	Were the study subjects and setting described in detail	Was data analysis conducted with sufficient sample coverage	Were valid methods used to identify condition	Was the condition measured in a standard and reliable way	Was the statistical analysis appropriate	Was the response rate adequate, and if not, was the low response rate managed appropriately
**Alice Guan et al. (2019)**	Yes	Yes	Yes	Yes	Yes	Yes	Yes	Yes	Not Applicable
**Mário Círio Nogueira et al. (2018)**	Yes	Yes	Yes	Yes	Yes	Yes	Yes	Yes	Not Applicable
**Li Tao et al. (2015)**	Yes	Yes	Yes	Yes	Yes	Yes	Yes	Yes	Not Applicable
**Kirsten Y. Eom et al. (2023)**	Yes	Yes	Yes	Yes	Yes	Yes	Yes	Yes	Not Applicable
**Joe Feinglass et al. (2015)**	Yes	Yes	Yes	Yes	Yes	Yes	Yes	Yes	Yes
**Nelson Luiz Renna Junior et a. (2021)**	Yes	Yes	Yes	No	Yes	Yes	Yes	Yes	Yes

Table S2. Methodological Assessment Using Crombie's Criteria.

Author (Year)	Clarity of the research question	Appropriate population selection	Reliable measurement of variables	Adequate control for confounding variables	Use of appropriate statistical methods	Comprehensive description of the study setting	External validity of findings	Consistency of results	Reproducibility of the study
**Taylor Anderson (2023)**	Yes	Yes	Yes	No	Yes	Yes	Partially Met	Yes	Yes
**Tianhui Chen et al. (2023)**	Yes	Yes	Yes	No	Yes	Yes	Yes	Yes	Yes
**Arash Azin et al. (2022)**	Yes	Yes	Yes	Partially Met	Yes	Yes	Partially Met	Yes	Yes
**Fei Wang et al. (2021)**	Yes	Yes	Yes	Yes	Yes	Yes	Partially Met	Yes	No
**Tomi F. Akinyemiju et al. (2015)**	Yes	Yes	Yes	Yes	Yes	Yes	Partially Met	Yes	Partially Met
**Tomi Akinyemiju et al. (2021)**	Yes	Yes	Yes	Partially Met	Yes	Yes	Partially Met	Yes	Partially Met

Table S3. Methodological Quality of Cohort Studies (JBI Checklist).

Author (Year)	Were the two groups similar and recruited from the same population?	Were exposures measured similarly to assign people to both exposed and unexposed groups?	Was the exposure measured in a valid and reliable way?	Were confounding factors identified and strategies to deal with them stated?	Were the groups/participants free of the outcome at the start of the study?	Were the outcomes measured in a valid and reliable way?	Was the follow-up time reported and sufficient to detect an outcome?	Was follow-up complete, and if not, were differences between groups in terms of follow-up adequately described and analyzed?	Were strategies to address incomplete follow-up utilized?	Was appropriate statistical analysis used?
**Alexander Boyko et al. (2023)**	Yes	No	Yes	Partially Met	Yes	Yes	Yes	Yes	Yes	Yes
**Oluwole A. Babatunde et al. (2021)**	Yes	Yes	Yes	Yes	Yes	Yes	Yes	Yes	Yes	Yes
**Maureen Sanderson et al. (2015)**	Yes	Yes	Yes	Yes	Yes	Yes	Yes	Yes	Yes	Yes
**Javaid Iqbal et al. (2015)**	Yes	Yes	Yes	Yes	Yes	Yes	Yes	Yes	Yes	Yes
**Esther M. John et al. (2021)**	Yes	Yes	Yes	Yes	Yes	Yes	Yes	Yes	Yes	Yes
**Salma Shariff-Marco et al. (2015)**	Yes	Yes	Yes	No	Yes	Yes	Yes	Yes	Yes	Yes
